# NaOH as an Aqueous Electrolyte to Improve the Performance of Electric Double-Layer Capacitors—A Molecular Dynamics Study

**DOI:** 10.3390/nano15090649

**Published:** 2025-04-25

**Authors:** Lifeng Ni, Jin Yu

**Affiliations:** Shanghai Key Laboratory of Mechanics in Energy Engineering, School of Mechanics and Engineering Science, Shanghai Institute of Applied Mathematics and Mechanics, Shanghai University, Shanghai Frontier Science Center of Mechanoinformatics, Shanghai 200072, China; nlf@shu.edu.cn

**Keywords:** NaOH electrolyte, electric double layer, supercapacitors

## Abstract

Aqueous electrolytes are widely used in supercapacitors (SCs) because of their high stability, wide voltage window, and safety features at elevated temperatures. Among alkaline electrolytes, KOH is most commonly used, and other electrolytes are less addressed. In this work, we meticulously investigated the diffusion behavior of Na^+^ and K^+^ in aqueous electrolytes going through hierarchical porous activated carbon materials by employing molecular dynamic simulations. Our results show that the diffusion coefficient of NaOH is much larger than that of KOH under different concentrations, electric fields, and temperatures. We attributed this to the radical of ions going through the mesopores with layered structures. The advantage of high diffusion and low cost of NaOH electrolyte suggests that it could be a potential candidate to improve the performance of SCs.

## 1. Introduction

With the extensive use of fossil fuels, environmental issues caused by carbon accumulation and carbon dioxide emissions are becoming increasingly severe. As a result, renewable energy has emerged as the most promising alternative to petrochemical fuels [1,2]. The improvement of renewable energy further promotes the development of reliable electrochemical storage technologies like batteries, fuel cells, and supercapacitors (SCs). Especially, SCs are gaining attention over batteries due to their rapid charging rate (1–10 s) and high cyclic stability (over 30,000 h) [3,4]. The charge storage and capacitance of SCs are primarily determined by the electrode and electrolyte materials [5,6]. Developing new electrode materials, combined with proper electrolytes to achieve higher capacitance and better performance, is a primary goal in the development of SCs [7,8].

The latest advances in electrode materials involve carbon-based materials, metal oxides, and conducting polymers. Due to the low manufacturing cost, high thermal and chemical stability, and excellent electrical conductivity, carbon-based electrode materials are highly favored in energy storage devices among all advanced electrode materials [9]. The high specific surface area of carbon materials enhances the capacitance of Electric Double-Layer Capacitors (EDLCs) [10], making them ideal for use in SCs. Activated carbon (AC) is one of the most promising materials among carbon-based electrode materials due to its extensive surface area, excellent electrical performance, and cost-effectiveness. Recent advancements have shown that the surface area of AC reaches up to 3000 $m^{2}\cdot g^{-1}$ and its electrochemical properties, including capacitance, time constant, energy density, and conductivity, are enhanced dramatically [11,12].

On the other hand, electrolytes play a crucial role in the performance of SCs, but a perfect electrolyte has yet to be developed [13]. The electrolyte is essential in forming EDLCs and facilitating reversible redox reactions for pseudocapacitors. Compared with organic electrolytes, aqueous electrolytes exhibit potential applications due to their high chemical and thermal stability, wide operating voltage window, negligible vapor pressure, and nonflammability [14]. Among various aqueous electrolytes, KOH solution is the most widely used alkaline electrolyte. It offers an ionic conductivity similar to that of H_2_SO_4_, ensuring high power output for SCs; its wide electrochemical window aids in enhancing the energy density of capacitors, ensuring the operational voltage and overall energy storage capacity of SCs [15]; it also possesses good chemical stability and compatibility with electrode materials, ensuring the long-term cycling stability of the SCs [16,17]. Solid electrolytes enable the development of miniaturized and ultra-thin supercapacitors (SCs) due to their reliability, high specific energy, and freedom from electrolyte leakage. However, their practical application in SCs faces several challenges, including the low ionic conductivity of most polymer electrolytes at room temperature, limited solubility of electrolyte salts in the polymer matrix, and poor interfacial contact between the electrolyte and electrodes [18,19].

In a very recent study, Li et al. utilized the Fenton chemistry concept to achieve hierarchical porous biomass-activated carbon material FHWSAC-3, which boasts a significantly high specific surface area of 3440 $m^{2}\cdot g^{-1}$ and double activation efficiency compared to traditional methods [20]. In that work, the authors built a physical model of FHWSAC-3 based on experimental data and confirmed through molecular dynamics (MD) simulations that the structure facilitates rapid ion diffusion in KOH electrolyte, aligned with excellent electrochemical performance. The excellent electrochemical performance, combined with low pollution and carbon emissions during production, makes FHWSAC-3 promising for SCs. However, the cost of KOH limits its application in industry. Exploring low-cost aqueous electrolytes with good conductivity, wide electrochemical window, excellent chemical stability, and good compatibility with electrode materials [21,22] becomes an important topic in SCs.

Studies have shown that in aqueous electrolytes, the smaller the size of the cation, the greater the specific capacitance of the SCs [23,24]. In this work, we performed numerical simulations on the diffusion properties of ions passing through FHWSAC-3 to explore the feasibility of NaOH solution as a potential electrolyte. Our results show that under various concentrations, electric fields, and temperatures, the diffusion coefficient of Na^+^ is larger than that of K^+^, suggesting that NaOH may exhibit superior electrochemical performance in FHWSAC-3. Current research underscores the importance of electrolyte selection, demonstrating that aqueous NaOH electrolyte is a potential candidate for EDLCs with low cost.

## 2. Model and Methodology

### 2.1. Physical Model

**Figure 1:**
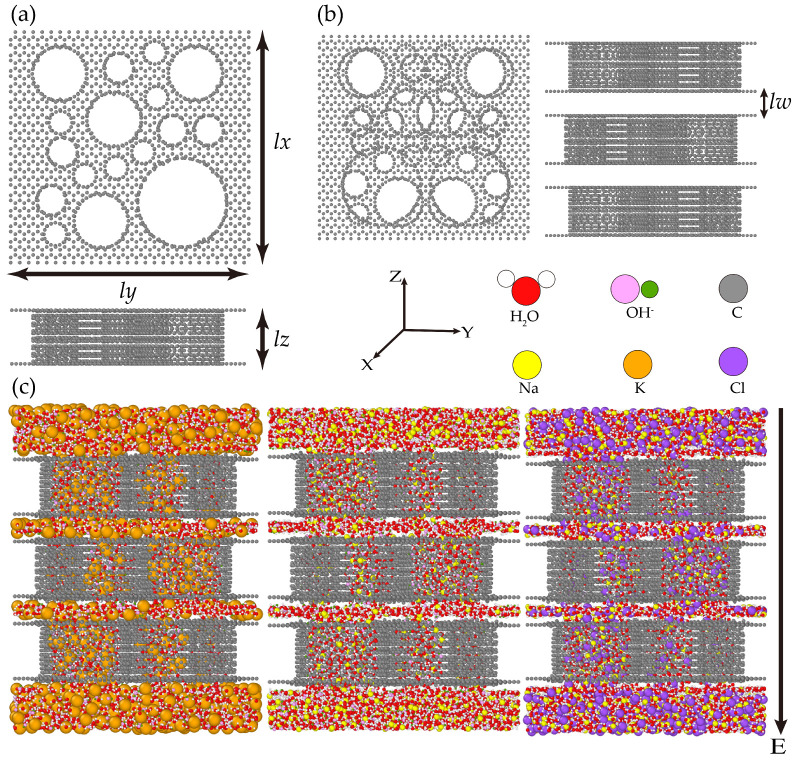
Schematic of the EDLCs model. (**a**) Top and front views of the single carbon framework. (**b**) Top and front views of the FHWSAC-3 hierarchical structure. (**c**) Snapshot of KOH, NaOH, and NaCl going through FHWSAC-3.

As shown in Figure 1, the EDLCs in the current work are modeled by stacking three layers of carbon framework with an interlayer space lw = 7.5 Å. Each layer of the carbon framework is a random cylindrical pore model oriented along the Z direction with the geometric dimensions lx×ly×lz being 63.10 Å× 65.08 Å× 15.00 Å, which is in agreement with the experimental pore volume and pore size distribution of the FHWSAC-3 hierarchical structure [20]. And three types of solutions—KOH, NaOH, and NaCl—were chosen to study the transport properties of ions, as shown in Figure 1c. The number of water molecules in each solution is 6256, while the numbers of Na^+^, K^+^, Cl^−^, and OH^−^ are determined by the concentration of the solution.

### 2.2. Force Field

The TIP4P/2005 model, which is extensively used in computational chemistry and molecular dynamics simulations [25], was adopted to represent the behavior of water molecules. The Madrid-Transport and Madrid-2019 force fields were used to calculate the diffusion of Na^+^, K^+^, Cl^−^ in the electrolyte [26,27]. The force field of OH^−^ was utilized following previous work [28]. The interaction between carbon atoms within the carbon framework was described by the AIREBO potential. In addition to the interactions between carbon atoms, the interactions between various atoms were calculated by the Lorentz–Berthelot mixing rule. The detailed force field parameters are listed in Table 1.

**Table 1:** Force field parameters for C, Na^+^, K^+^, Cl^−^, OH^−^.

Atom	M (g/mol)	q (e)	$\sigma_{i}$ (Å)	$\epsilon_{i}$ (eV)	$\sigma_{{\mathrm{MO}}_{w}}$ (Å)	$\epsilon_{{\mathrm{MO}}_{w}}$ (eV)
C	12.0107	0.0000	3.4000	0.00373	3.1900	0.00406
Na(NaOH)	22.9898	0.7500	2.2174	0.01526	2.6084	0.00822
Na(NaCl)	22.9898	0.8500	2.2174	0.01526	2.3872	0.00822
K	39.0983	0.7500	2.3014	0.02058	2.8954	0.01451
H	1.00794	0.4681	1.4430	0.00191	2.3009	0.00391
O	15.9994	−1.2181	3.6500	0.00260	3.4044	0.00457
Cl	35.4529	−0.8500	4.6991	0.00080	3.9290	0.00253

M refers to the C, Na, K, Cl, O, and H, $O_{w}$ refers to the O-atom of water (TIP4P/2005 model [25]). In order to obtain a realistic contact angle between water and graphite, the force field parameters that define the interaction between C and O of water molecules have been modified [29].

### 2.3. MD Simulations

Our MD simulations were carried out using the Large-scale Atomic/Molecular Massively Parallel Simulator (LAMMPS) [30]. The equations of motion of the system were solved by the Verlet algorithm [31] with a time step of 0.0005 ps. Periodic boundary conditions were imposed along every direction. The SHAKE algorithm [32,33] was employed to keep specific geometric constraints during the simulation. For water molecules, the bond length of OH was set to be 0.96 Å, and the bond angle of H–O–H was constrained to be $104.52^{\circ }$ [25]. For OH^−^, the bond length was fixed as 0.98 Å [28]. To achieve a rigid configuration for the carbon frameworks in the simulation, the fix-rigid command in LAMMPS was utilized [34]. And the cutoff radius for L-J potential was set to 12 Å, and the cutoff radius for Coulomb interactions was established at 10 Å. Long-range electrostatic interactions are handled by the Particle–Particle Particle–Mesh (PPPM) method [35] with a relative error of $10^{-5}$.

**Figure A1:**
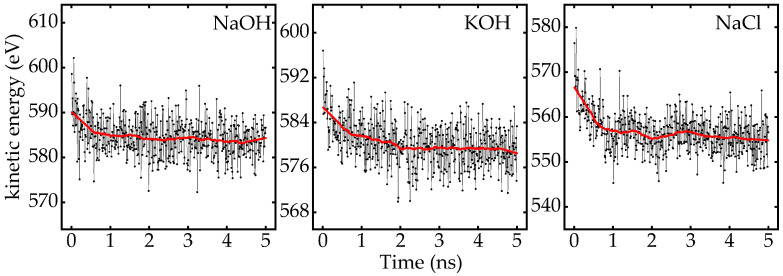
Kinetic energy of NaOH, KOH, and NaCl with a concentration of 6 mol/L under an electric field of 0.5 V/Å at 300 K. The red line in the figure is the result of smoothing using the Savitzky–Golay filter.

The EDLCs underwent an initial relaxation in the NPT ensemble with a pressure of 1 atmosphere applied along the Z-axis, lasting for 5 ns. Then, an electric field along the negative Z-axis was applied for the NVT ensemble as shown in Figure 1. In the first 2 ns, the kinetic energy of the system showed significant fluctuations. We attribute this to the external field which disrupts the relaxation equilibrium of the system [see Figure A1 in Appendix A.1]. After 2 ns of relaxation, the mean square displacement (MSD) of Na^+^ and K^+^ was calculated. The Nosé–Hoover thermostat and barostat [36,37] were used with relaxation times of 0.1 ps and 1 ps, respectively, to maintain the temperature and pressure during the simulation. Kamberaj’s adaptations [38] to the Nosé–Hoover thermostat are implemented for rigid bodies. This study builds upon our previous work, in which the computed MSDs for KOH showed good agreement with experimental results [20] and we believe that prior validation would offer a solid foundation for the present investigation.

## 3. Result and Discussion

### 3.1. Effect of Concentration on the Diffusion of Na^+^ and K^+^

**Figure A2:**
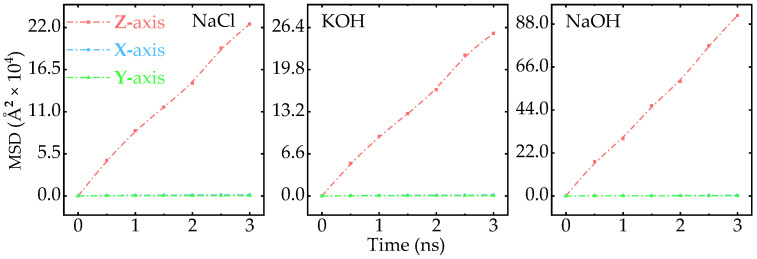
MSD of ions in NaCl, KOH, and NaOH solutions along different axis.

Ion concentration of electrolytes is a crucial factor in determining the electrochemical performance of SCs [39]. To study the effect of ion concentration on the EDLCs based on FHWSAC-3, we started our numerical simulation by setting the ion concentration of different solutions to 6 mol/L, 4 mol/L, and 2 mol/L, respectively. To model the experimental environment, the temperature was set to 300 K. Driven by a vertical electric field of 0.5 V/Å, ions passed through FHWSAC-3. And the MSD was calculated to evaluate the performance of various ions. Herein, we only study the MSD along the Z-axis under the external electric field, as the MSD along the X- and Y-axis can be neglected [see Figure A2 in Appendix A.2].

**Figure 2:**
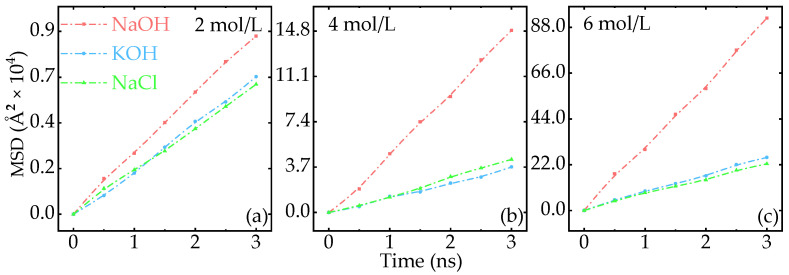
Concentration-dependent MSD in different solutions. (**a**–**c**) Represent the MSD with a concentration of 2 mol/L, 4 mol/L, and 6 mol/L, respectively. The pink, light blue, and green solid-dash lines stand for NaOH, KOH, and NaCl, respectively.

**Figure A3:**
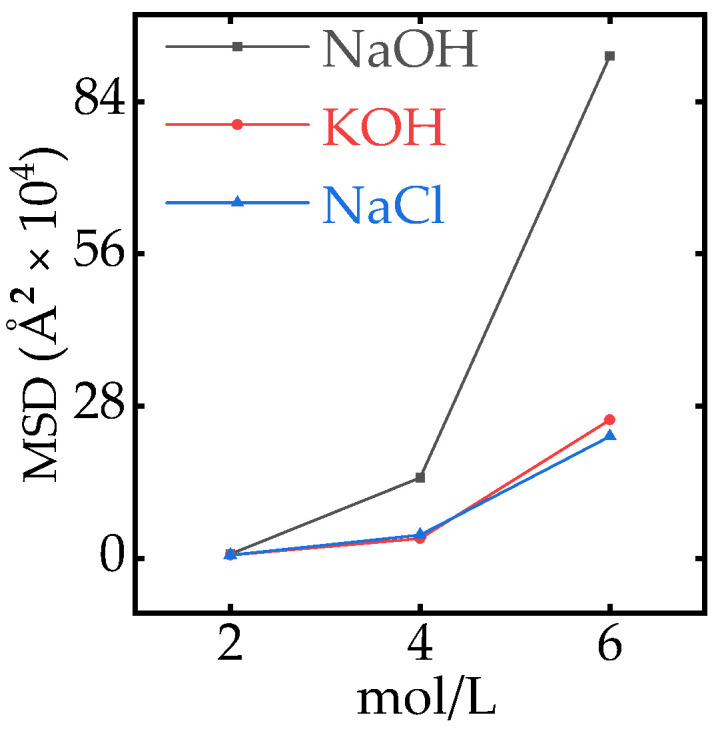
Snapshot of the MSD for NaOH, KOH, and NaCl at 3 ns with the aqueous concentration being 2 mol/L, 4 mol/L, and 6 mol/L, respectively.

**Figure A4:**
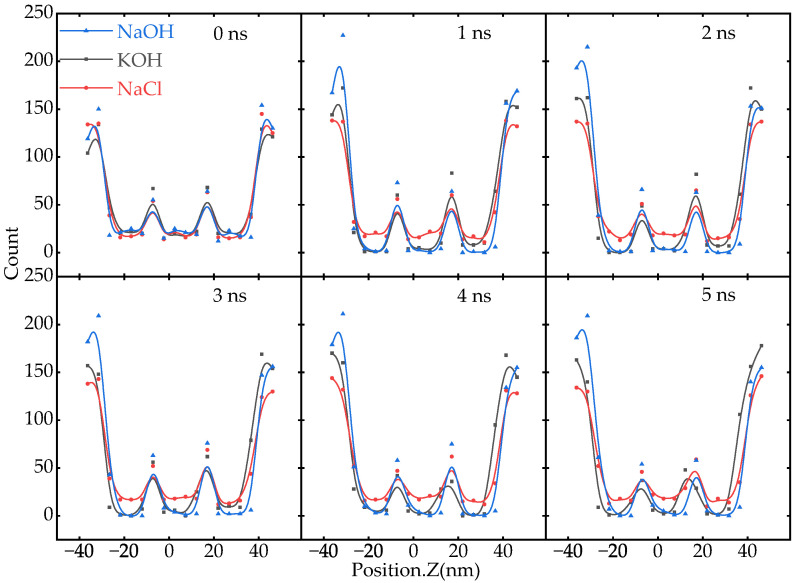
Mass density profile of NaOH, KOH, and NaCl along the Z-axis with a concentration of 6 mol/L under an electric field of 0.5 V/Å at 300 K.

As shown in Figure 2, the calculated MSD of various ions increases as the ion concentration increases, which is consistent with other reports. At a low concentration of 2 mol/L, the MSDs of NaCl and KOH are very close, while the value of NaOH is higher. Accordingly, the calculated diffusion coefficient of NaOH is 1.29 times larger than that of the other two. Snapshot of MSD at 3 ns [see Figure A3 in Appendix A.3] shows that when the ion concentration increases to 4 mol/L, the MSD of NaOH is $14.8\times 10^{4}$${Å}^{2}$, which is 16.5 times that at the low concentration case. In contrast, the MSD of NaCl and KOH increases by a factor of 5.2. Thus, the change of MSD in NaOH shows a distinct superiority, far surpassing the changes in the other two solutions. The calculated diffusion coefficient shows that the value of NaOH is 4.2 times that of NaCl and KOH, indicating that the ion transport performance of NaCl and KOH is inferior to NaOH. When the concentration further increases to 6 mol/L, the calculated MSD of NaOH reaches up to $92.4\times 10^{4}$${Å}^{2}$, whereas the values of KOH and NaCl are $25.5\times 10^{4}$${Å}^{2}$ and $22.4\times 10^{4}$${Å}^{2}$, respectively, rendering a much larger gap. Moreover, snapshots of the mass density profile at different times reveal that ions in FHWSAC-3 are nearly the same [see Figure A4 in the Appendix A.4], suggesting continuous transportation of ions in the tunnel. To this end, we conclude that NaOH has the highest ion diffusion rate at all concentrations. Consistent with our simulation, experiments show that using NaOH instead of KOH would significantly improve the performance of SCs [see Table A1 in Appendix A.5]. Typically, the MSD of NaOH increases by two orders of magnitude when the concentration increases from 2 mol/L to 6 mol/L. Thus, EDLCs using NaOH as an electrolyte may exhibit superior electrochemical performance to those using KOH or NaCl in a wide range of solution concentrations.

### 3.2. Effect of Electric Field on the Diffusion of Na^+^ and K^+^

Concentration is one of the important variables affecting the transport performance of ions, while the driving force of the EDLCs originates from the external electric field applied along the electrodes. Thus, electric field strength is a key factor in determining the transport properties of ions. Previous simulations have shown that a solution with a concentration of 6 mol/L exhibits the largest diffusion coefficient. Therefore, when investigating the effect of the electric field on the transport properties of ions, we set the ion concentration to 6 mol/L and the temperature to room temperature. A series of electric field strengths ranging from 0.05 V/Å to 0.5 V/Å was set to investigate the diffusion behavior of different ions within the electrodes.

**Figure 3:**
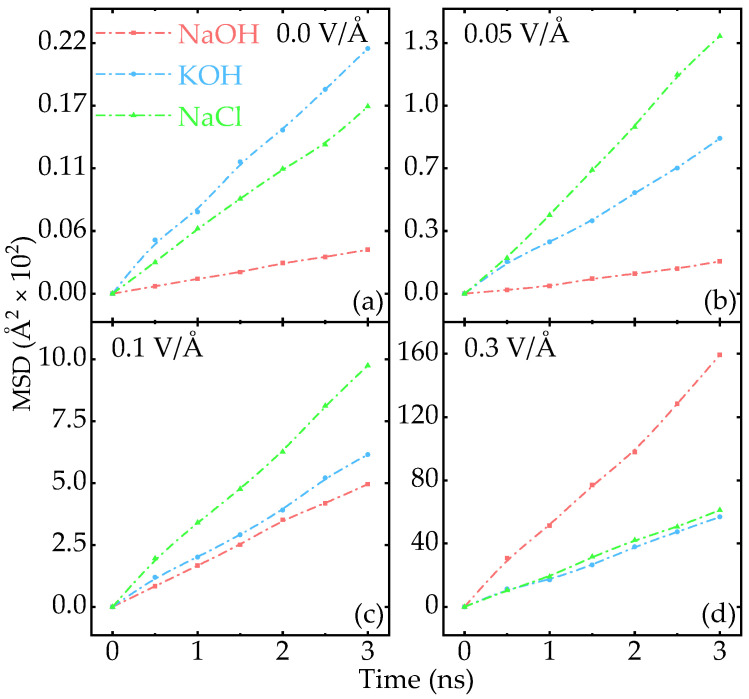
Electric field-dependent MSD in different ion solutions. (**a**–**d**) represent the MSD of three electrolytes under increasing electric field of 0.0 V/Å, 0.05 V/Å, 0.1 V/Å, and 0.3 V/Å, respectively.

**Figure A5:**
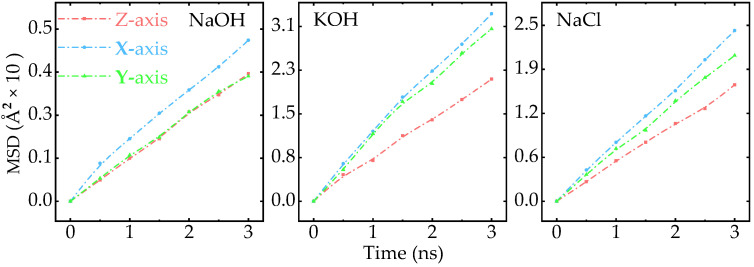
The MSD of ions in NaOH, KOH, and NaCl solutions along different axis without the external field.

**Figure A6:**
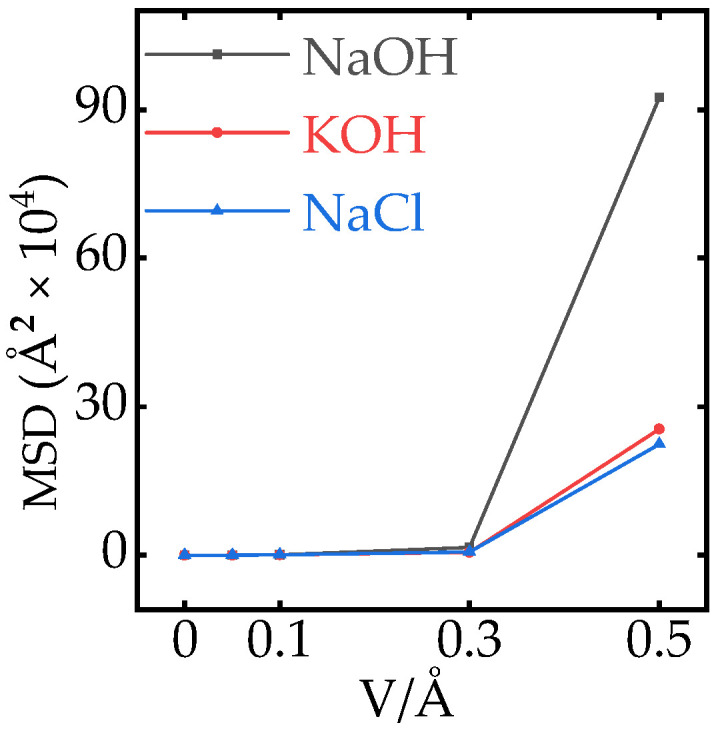
Snapshot of the MSD for NaOH, KOH, and NaCl at 3 ns with the external electric field strength being 0 V/Å, 0.05 V/Å, 0.1 V/Å, 0.3 V/Å, 0.5 V/Å, respectively.

The calculated MSDs of NaOH, KOH, and NaCl are shown in Figure 3, respectively. According to Kumar and Yashonath, K^+^ is more mobile than Na^+^ due to the strong hydration around Na^+^ in the case of self-diffusion [40]. Our results show a similar trend [see Figure A5 in Appendix A.6] that the MSD of K^+^ is larger than that of Na^+^ without an external field. Since the horizontal plane is not hindered by the framework, the MSD along the in-plane direction is slightly larger than that along the Z-axis. Once an external electric field is applied, the diffusion of Na^+^ gradually surpasses that of K^+^. Due to the complexity of the hydration of Na^+^ and K^+^, we currently attribute this to the combination of the atomic mass of ions, confinement effects in the pore structure, and the hydration shell distortion [41]. Similar to the effect of concentration, the MSD of all solutions increases when the electric field strength increases and the trend of the curvature for KOH and NaCl are very close [see Figure A6 in Appendix A.7]. Especially, the MSD of NaOH exhibits a remarkable response to the external electric field. Under a low electric field of 0.05 V/Å, the MSD of NaCl is the highest, that is calculated to be 135.6 ${Å}^{2}$, approximately 10 times that of NaOH. As shown in Figure 3c, when the electric field strength increases to 0.1 V/Å, the MSD of all three solutions increases slightly and NaCl still holds an advantage. The MSD of ions in NaCl is about two times that of NaOH and KOH, suggesting that NaCl may be a good candidate for the electrolyte of EDLCs under low electric fields. As the electric field strength increases, the trend of the calculated MSD for NaCl and KOH varies slightly, while the curvature of NaOH exhibits a sharp response. As the strength of the external electric field further increases, the strong hydration around Na^+^ is gradually counteracted by the electric field [41], resulting in Na^+^ diffusing more rapidly than K^+^ in the FHWSAC-3. When the electric field strength increases to 0.3 V/Å, the calculated MSD of NaOH surpasses the other two, which is 4 times that of KOH and NaCl. The advantage of NaOH is further established when the external electric field increases to 0.5 V/Å as shown in Figure 2c. Considering the fact of high ionic transport performance and low-cost production, NaOH would be an alternative to KOH as an electrolyte in EDLCs.

### 3.3. Effect of Temperature on the Diffusion of Na^+^ and K^+^

Temperature regulation is essential for sustaining the optimal performance of SCs. To evaluate the performance of the proposed electrolyte in the actual working environment, we performed additional calculations to study the effect of temperature on ionic transportation. Herein, the FHWSAC-3 was embedded in an electrolyte with the concentration being 6 mol/L. And the temperature varied from 250 K to 350 K, which lies in the extreme temperature range of common aqueous electrolytes. Considering that the MSD under high electric fields far exceeds that under low electric fields, the external electric field used in the simulation was set to be 0.5 V/Å.

**Figure 4:**
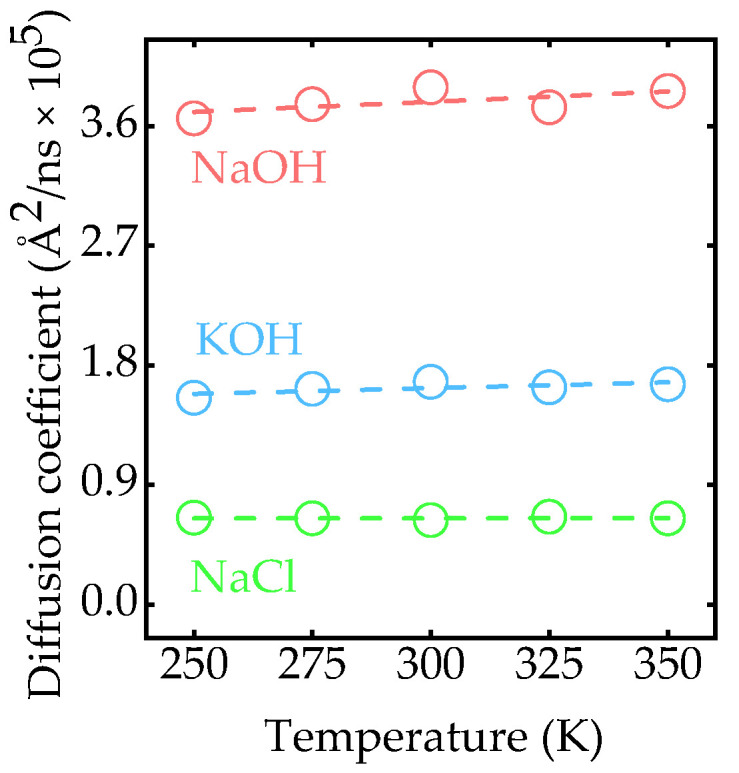
Temperature-dependent diffusion coefficient of different electrolytes.

Different from organic electrolytes, the ion diffusion coefficient [42] of NaOH, KOH, and NaCl remains in a stable state in a wide temperature window. As shown in Figure 4, when the temperature increases from 250 K to 350 K, the coefficient of NaOH and KOH increases from $3.7\times 10^{5}$${Å}^{2}/\mathrm{ns}$ and $1.6\times 10^{5}$${Å}^{2}/\mathrm{ns}$ to $3.9\times 10^{5}$${Å}^{2}/\mathrm{ns}$ and $1.7\times 10^{5}$${Å}^{2}/\mathrm{ns}$, respectively. On the contrary, the trend of the diffusion coefficient for NaCl is nearly horizontal, indicating that ionic transport in NaCl solution is robust against temperature. In all three solutions, the effect of temperature on the ionic diffusion coefficient is so weak that it is negligible. However, the diffusion coefficient of NaOH exhibits an advantage over the other two. From 250 K to 350 K, the calculated diffusion coefficient of NaOH is approximately twice that of KOH and four times that of NaCl. From the aspect of stability and ionic transport performance, NaOH could be a very good substitute for KOH in EDLCs.

## 4. Conclusions

In general, MD simulations were conducted to study the ionic diffusion behavior of NaOH, KOH, and NaCl in FHWSAC-3, to improve the performance of EDLCs from theoretical investigation. Due to the existence of mesopores, ion diffusion is enhanced in activated carbon materials. In particular, NaOH consistently demonstrates a much larger MSD compared to KOH and NaCl under various concentrations, electric field strengths, and temperatures. Moreover, the diffusion performance of NaOH remains stable in a wide temperature window from 250 K to 350 K, further emphasizing its advantage over some organic and ionic liquid electrolytes. These results suggest that Na^+^-based electrolytes, due to their high diffusion efficiency and cost-effectiveness, could be a potential candidate for EDLCs.

## Data Availability

Data are contained within the article.

## Abbreviations

The following abbreviations are used in this manuscript:

SCs	supercapacitors
EDLCs	Electric Double-Layer Capacitors
AC	Activated carbon
MD	molecular dynamics
LAMMPS	Large-scale Atomic/Molecular Massively Parallel Simulator
PPPM	Particle–Particle Particle–Mesh
MSD	mean square displacement

## Appendix A. Supplementary Information

### Appendix A.1. Kinetic Energy of System

During the initial 2 ns, notable fluctuations occur in the kinetic energy of the system. This behavior can be explained by the external field disturbing the system’s equilibrium state. As the system re-establishes equilibrium, the kinetic energy stabilizes after 2 ns. The diffusion coefficient presented in the manuscript was obtained by fitting the MSD data after 2 ns, as shown in Figure A1.

### Appendix A.2. MSD of Ions Along Different Axis

As shown in Figure A2, the MSD along X- and Y-axes is negligible compared to that along Z-axis, so all the discussion in the manuscript is based on the result of MSD along Z-axis. The concentration of the solution was set to be 6 mol/L and the external electric field is 0.5 V/Å.

### Appendix A.4. Mass Density Profile of Ions

Snapshots of the data for the mass density profile along the Z-axis are provided in Figure A4. Herein, the accumulation of ions occurs at the edge and interface of the carbon frameworks, while ions in the tunnel are very limited.

### Appendix A.5. Comparison of Carbon-Based SCs with Different Electrolytes

**Table A1:** Maximum specific capacitance of carbon-based supercapacitors with aqueous electrolyte.

Material Used	Measurement Protocol	Electrolyte	Maximum Specific Capacitance
Graphene	GCD (1 $A\cdot g^{-1}$)	3 mol/L NaOH	596 $F\cdot g^{-1}$ [18]
Graphene	GCD (1 $A\cdot g^{-1}$)	6 mol/L KOH	355 $F\cdot g^{-1}$ [43]
Carbon	GCD (1 $A\cdot g^{-1}$)	6 mol/L KOH	331 $F\cdot g^{-1}$ [44]
Carbon	GCD (1 $A\cdot g^{-1}$)	6 mol/L KOH	222.92 $F\cdot g^{-1}$ [45]

The maximum specific capacitance of NaOH with a concentration of 3 mol/L is much larger than the other electrolytes, suggesting potential applications in carbon-based SCs.

### Appendix A.6. Self-Diffusion of Ions in FHWSAC-3

When there is no electric field, ions prefer to diffuse isotropically even in the layered structure with “nano holes”. As shown in Figure A5, the MSDs for specific solution along X-, Y- and Z-axes are nearly the same, indicating the case of self-diffusion in bulk solutions.
